# Supplementary material for: Mitochondrial decline in the ageing old world primate retina: Little evidence for difference between the centre and periphery
Source: PLoS One. 2023 May 2;18(5):e0273882. doi: 10.1371/journal.pone.0273882 (PMC10153720; doi:10.1371/journal.pone.0273882)
Supplement: S1 File — (DOCX) [file pone.0273882.s001.docx]

**Mitochondrial decline in the ageing old world primate retina: little evidence for difference between the centre and periphery**

Uncropped Western blots. For figures


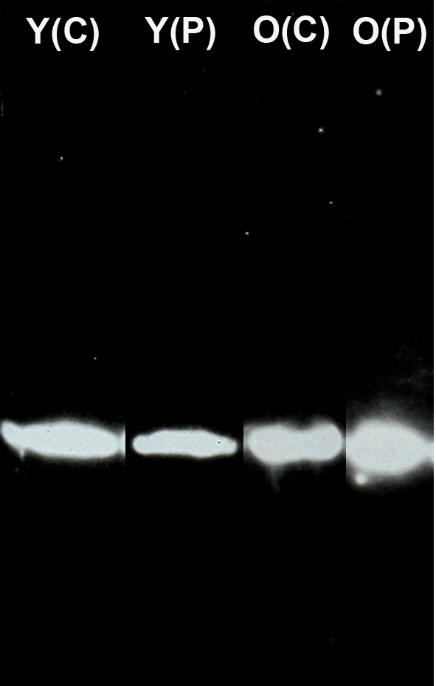

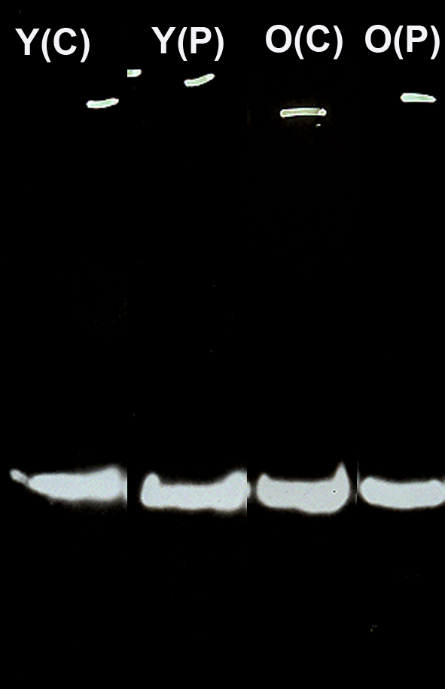


Figure 1. Uncropped Westerns for complex II (left) and for related GAPDH (right).

Abbreviations Y Young, O Old, C centre, P periphery. See methods for details.


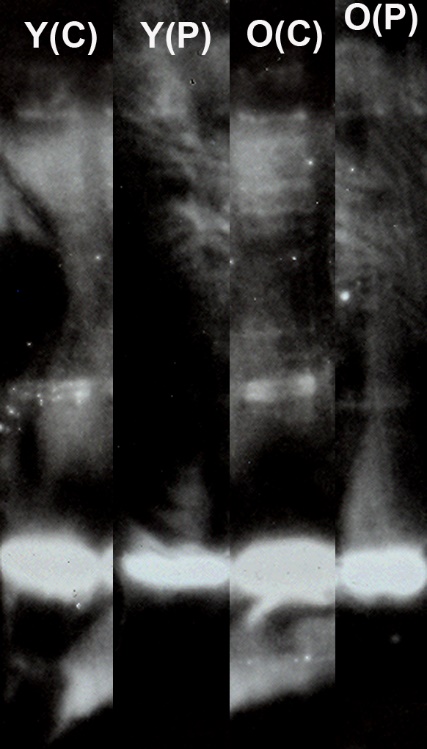

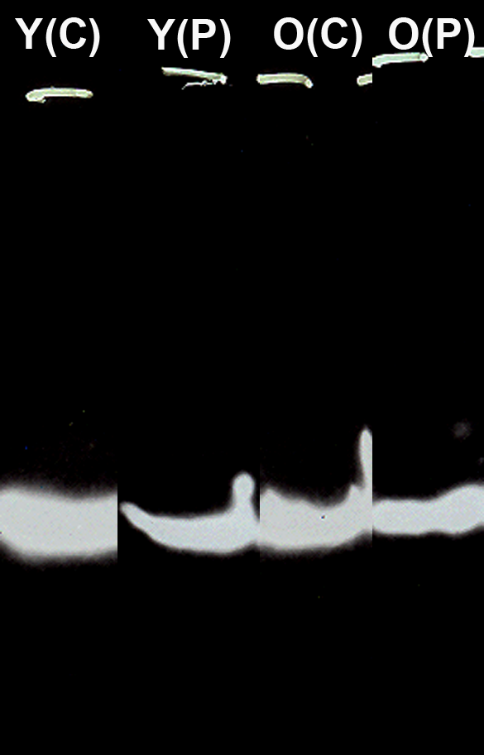


Figure IV. Uncropped Westerns for complex IV (left) and related GAPDH (right)

Abbreviations Y Young, O Old, C centre, P periphery. See methods for details.


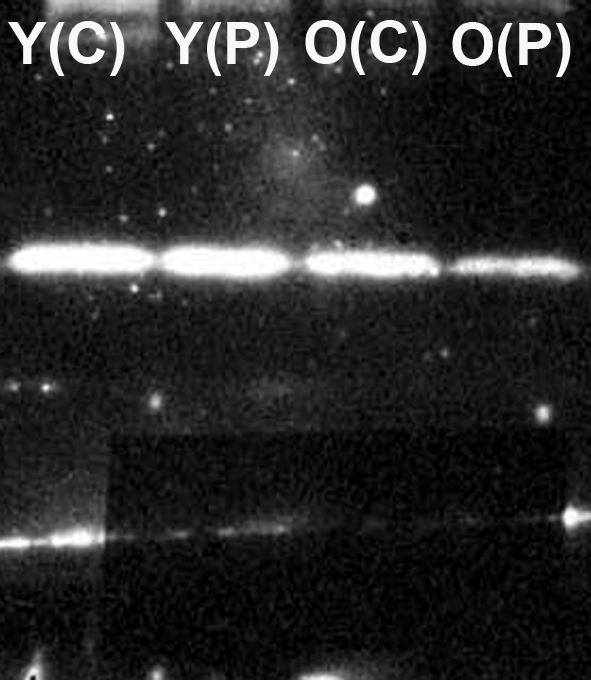

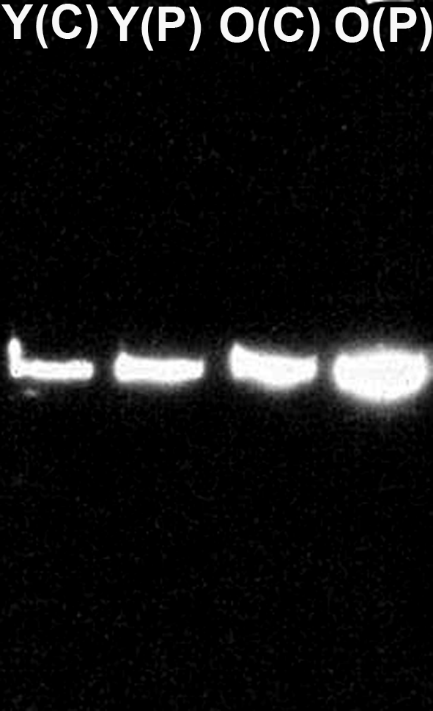


Figure 5A. Uncropped Westerns for TOM20 (left) and related related beta actin (right)

Abbreviations Y Young, O Old, C centre, P periphery. See methods for details.


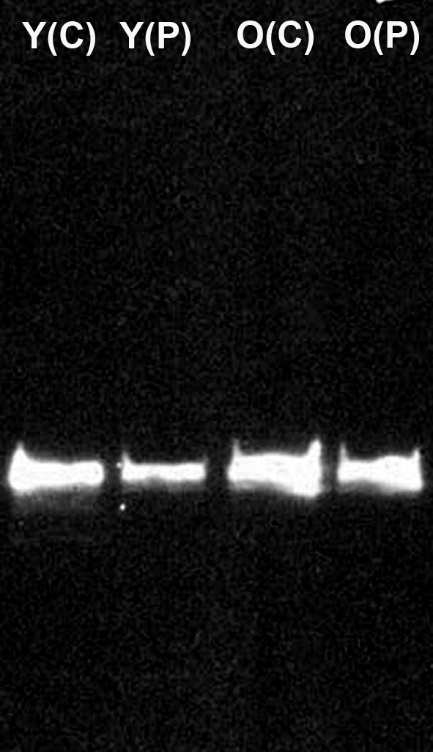

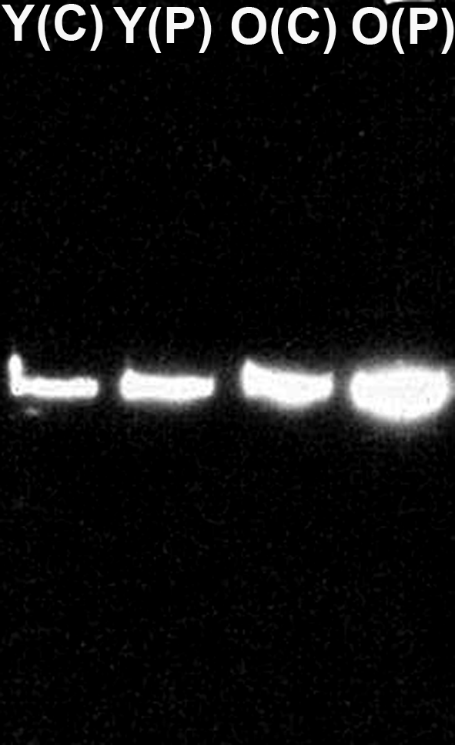


Figure 5B. Uncropped Westerns for VDAC (left) and related beta actin (right). Same as above.

Abbreviations Y Young, O Old, C centre, P periphery. See methods for details.
